# Transcriptomic and Metabolomic Profiling Identifies a Core Gene–Metabolite Axis Driving African Swine Fever Virus Replication in the Soft Tick *Ornithodoros lahorensis*

**DOI:** 10.1096/fj.202602001RR

**Published:** 2026-08-31

**Authors:** Jin Luo, Kaifei Guo, Fangyu Xiao, Ruoqi Wang, Junhui Guo, Qiaoyun Ren, Shuaiyang Zhao, Muhammad Kashif Obaid, Guiquan Guan, Hong Yin

**Affiliations:** ^1^ State Key Laboratory of Animal Disease Control and Prevention, Key Laboratory of Veterinary Parasitology of Gansu Province Lanzhou Veterinary Research Institute, Chinese Academy of Agricultural Sciences Lanzhou China; ^2^ College of Agriculture and Biological Sciences Yuxi Normal University Yuxi China; ^3^ College of Veterinary Medicine Hunan Agricultural University Changsha China; ^4^ College of Veterinary Medicine Gansu Agricultural University Lanzhou China; ^5^ Nanning Animal Disease Prevention and Control Center Nanning China; ^6^ Jiangsu co‐Innovation Center for the Prevention and Control of Important Animal Infectious Disease and Zoonosis Yangzhou University Yangzhou Jiangsu China

**Keywords:** African swine fever virus, metabolomics, *Ornithodoros lahorensis*, tick vector, transcriptomics, viral replication

## Abstract

African swine fever virus (ASFV) causes an incurable swine disease with nearly 100% mortality, posing a catastrophic threat to global pig production. The soft tick *Ornithodoros lahorensis* acts as a critical biological vector that sustains persistent ASFV replication and mediates long‐distance viral transmission, yet the molecular mechanisms governing ASFV‐tick interplay remain poorly understood. Here, we integrated transcriptomics and metabolomics to systematically dissect molecular changes in *O. lahorensis* across three infection stages: Uninfected control, early infection (7 days post‐infection, dpi), and late persistent infection (21 dpi). Multi‐omics integration revealed that ASFV extensively remodels tick host metabolism, predominantly activating purine/pyrimidine metabolism, lipid biosynthesis, and energy metabolism. We further characterized a conserved regulatory module consisting of 12 core genes and 8 signature metabolites that collectively support ASFV genome replication and virion assembly. Three hub metabolic genes (*TK1*, *ATP5F1B*, and *IMPDH*) were selected for functional validation via siRNA silencing in ticks; individual gene silencing suppressed ASFV loads by 89.2%, 91.5%, and 87.8%, respectively (*p* < 0.001***). This work represents the first comprehensive multi‐omics investigation of ASFV infection in *O. lahorensis* . We identified tick‐specific molecular targets to block vector‐mediated ASFV spread and established a standardized multi‐omics analytical pipeline for tick–virus interaction research. Our findings elucidate the mechanistic basis of long‐term ASFV persistence in soft ticks and deliver novel actionable clues for developing vector‐targeted ASF intervention strategies.

## Graphical abstract

Transcriptomic and metabolomic profiling of *Ornithodoros lahorensis* during African swine fever virus (ASFV) infection reveals that ASFV rewires host purine/pyrimidine metabolism, lipid metabolism, and energy metabolism. A core gene‐metabolite regulatory axis consisting of 12 key genes and 8 signature metabolites is identified to support viral genome replication and virion assembly. Functional validation demonstrates that three hub genes (TK1, ATP5F1B, and IMPDH) are essential for ASFV replication in the tick midgut, providing novel tick‐specific molecular targets to block vector‐mediated ASFV transmission and a universal multi‐omics framework for tick‐virus interaction research.

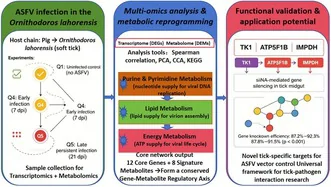

## 1 Introduction

African swine fever (ASF), triggered by the large double‐stranded DNA virus ASFV (family Asfarviridae), is a highly contagious transboundary lethal disease of suids [1]. Since its emergence in Kenya in 1921, ASF has spread across Africa, Europe, Asia, and the Americas, severely undermining global food security and swine production chains [2]. Distinct from most arboviruses, ASFV establishes lifelong persistent infection in soft ticks of the genus *Ornithodoros*, which serve as both natural viral reservoirs and transmission vectors. These ticks drive inter‐herd and cross‐regional viral circulation, underpinning the long‐term endemicity of ASF worldwide [3].

*Ornithodoros lahorensis* , a widespread soft tick across Eurasia, functions as a primary ASFV vector in Central Asia and fuels local and transboundary viral dissemination [4, 5]. Notably, ASFV replicates robustly in multiple tissues (midgut, salivary glands, Malpighian tubules) without inducing significant tick mortality, a trait that enables stable long‐term viral persistence and continuous transmission [6]. Nevertheless, how ASFV hijacks tick cellular components and metabolic pathways to sustain its replication remains poorly defined, creating a critical knowledge gap that restricts the development of vector‐based ASF control measures.

Integrated multi‐omics analysis has emerged as a powerful tool to untangle intricate pathogen‐host interactions in the post‐genomic era [7]. Combined transcriptomic and metabolomic profiling captures dynamic shifts in host gene expression and metabolite abundance upon infection, enabling high‐throughput identification of core regulatory molecules and functional networks linked to viral proliferation [8]. As a large DNA virus, ASFV is heavily dependent on host metabolic pathways to acquire nucleotides, lipids, and energy for genome duplication and virion morphogenesis [9]. Prior studies have confirmed that ASFV rewires purine/pyrimidine metabolism, glycolysis, and lipid synthesis in porcine macrophages, the primary mammalian host cells of ASFV [10]. However, it remains unknown whether ASFV employs identical metabolic manipulation strategies in soft ticks or evolves tick‐specific reprogramming patterns.

We hypothesized that ASFV infection triggers both conserved and tick‐specific metabolic remodeling in *O. lahorensis* , assembling a core gene–metabolite regulatory axis to maintain persistent viral replication. To test this hypothesis, we performed unbiased integrated transcriptomic and metabolomic sequencing on *O. lahorensis* at three representative ASFV infection stages. We applied Spearman correlation, principal component analysis (PCA), canonical correlation analysis (CCA), and KEGG enrichment to screen pivotal genes, metabolites, and metabolic pathways, followed by orthogonal validation via qRT‐PCR, targeted metabolomics, and in vitro siRNA interference in tick midguts. This study pursues four core objectives: (1) profile global transcriptomic and metabolomic responses of *O. lahorensis* to ASFV infection; (2) decode tick‐specific gene–metabolite networks that facilitate viral replication; (3) screen molecular targets to suppress ASFV persistence in soft ticks; (4) establish a universal multi‐omics workflow for tick–pathogen interaction research. Our work fills a major void in the molecular dissection of ASFV–soft tick interactions and provides innovative theoretical support for interrupting tick‐borne ASF transmission.

## 2 Materials and Methods

### 2.1 Ethics Statement

All animal experiments complied with the Animal Ethics Procedures and Guidelines of the People's Republic of China. The study protocol was approved by the Animal Administration and Ethics Committee of Lanzhou Veterinary Research Institute, CAAS (Approval No. LVRIAEC 2025–008).

### 2.2 Experimental Animals, Viruses and Ticks Sources

*Ornithodoros lahorensis* ticks were collected from an ASF‐free region in Xinjiang Uygur Autonomous Region, China. Species identity was verified via morphological observation and mitochondrial gene sequencing. Ticks were maintained in a climate‐controlled incubator (28°C, 85% relative humidity, 12 h light/12 h dark cycle). Zealand white rabbits served as blood‐feeding hosts to generate synchronized tick populations covering larvae, fourth‐instar nymphs, and adults with consistent physiological status. All ticks were starved for 7 days before infection to standardize feeding activity.

Six‐week‐old healthy pigs were purchased from an ASF‐negative commercial farm and acclimatized for 7 days in a biosafety Level 3 (BSL‐3) laboratory before use. The virulent Genotype II ASFV strain CN/SC/2019, endemic in China, was provided by Lanzhou Veterinary Research Institute, CAAS.

### 2.3 Tick and Pig Infection Assays & Sample Preparation

#### 2.3.1 ASFV Inoculation of Pigs

Experimental pigs were randomly allocated to infected and uninfected control groups (*n* = 3 per group). Infected pigs received an intramuscular injection of 2 mL ASFV suspension (10^6^ TCID_50_/mL); control pigs received an equal volume of sterile PBS (pH 7.4). All pigs were housed in independent negative‐pressure isolation units, with daily monitoring of ASF clinical signs (fever, anorexia, lethargy, skin erythema). Tick feeding was conducted 3–5 days post‐inoculation, when infected pigs displayed typical ASF symptoms and high peripheral blood viral loads (confirmed by qPCR).

#### 2.3.2 Tick Blood‐Feeding Infection

Starved ticks across all developmental stages were randomly split into two feeding cohorts with three biological replicates (100 mixed‐stage ticks per replicate). Cohort 1 fed on ASFV‐infected pigs with clinical symptoms; Cohort 2 fed on uninfected control pigs under identical conditions. Fully engorged ticks were immediately recovered and cultured under standardized incubator conditions for subsequent viral dynamic monitoring.

#### 2.3.3 Group Classification and Sample Collection

Engorged ticks were categorized into three experimental groups for multi‐omics and functional assays: Uninfected control (Q1), early infection (Q4, 7 dpi), and late persistent infection (Q5, 21 dpi). The two infection time points were defined based on ASFV viral load dynamics in *O. lahorensis* , tick persistent infection characteristics, and published criteria for staging ASFV infection in *Ornithodoros* ticks. At 7 dpi, ASFV initiates stable replication in tick midguts and hemolymph, representing early steady infection; at 21 dpi, viral replication peaks, with widespread viral distribution across salivary glands and other tissues, marking complete persistent infection [6].

Q1 ticks originated from ASF‐free pig feeding, with ASFV negativity validated via hemolymph qPCR at 4 days post‐feeding; Q4 and Q5 ticks were pathogen‐free individuals fed on infected pigs and harvested at 7 dpi and 21 dpi, respectively. Each biological replicate contained 50 pooled ticks (20 larvae, 20 nymphs, 10 adults) to guarantee sufficient RNA and metabolite yields and eliminate developmental stage bias. All samples were snap‐frozen in liquid nitrogen immediately after collection and stored at −80°C; repeated freeze–thaw cycles were strictly prohibited to prevent RNA and metabolite degradation.

#### 2.3.4 Quantification of ASFV Viral Load

Whole tick tissues were homogenized in sterile PBS, centrifuged to remove debris, and viral genomic DNA was extracted using a commercial nucleic acid purification kit. ASFV load was quantified via TaqMan qPCR targeting the B646L gene with published specific primers and probe [11]. The 20 μL reaction system contained 10 μL 2× TaqMan Master Mix, 0.4 μM forward/reverse primers, 0.2 μM probe, and 2 μL template DNA. Thermal cycling parameters: 95°C for 3 min, followed by 40 cycles of 95°C for 15 s and 60°C for 1 min. A standard curve was generated using 10‐fold serially diluted B646L plasmid (R^2^ = 0.998, amplification efficiency = 96.7%). Viral positivity was judged based on cycle threshold (Ct) values.

### 2.4 Transcriptomic Sequencing and Data Processing

#### 2.4.1 RNA Extraction and Library Construction

Total RNA was extracted from tick homogenates using the RNeasy Mini Kit (Qiagen, Germany), with on‐column DNase I digestion to eliminate genomic DNA contamination. RNA purity was determined via NanoDrop 2000 (A260/A280 = 1.8–2.1, A260/A230 > 1.7). RNA integrity was assessed on an Agilent 2100 Bioanalyzer; only samples with RIN ≥ 7.0 and 28S/18S rRNA ratio ≥ 1.5 were retained for library construction.

RNA‐seq libraries were built using the NEBNext Ultra RNA Library Prep Kit for Illumina. Briefly, mRNA was enriched with oligo(dT) magnetic beads, fragmented into 200–300 bp inserts, reverse‐transcribed with random hexamers, and subjected to second‐strand cDNA synthesis, end repair, dA‐tailing, and adapter ligation. PCR‐amplified libraries were quantified with a Qubit 4 Fluorometer, and insert size distribution (220–280 bp peak) was validated via Agilent 2100 before equal‐molar pooling for sequencing.

#### 2.4.2 Sequencing and Bioinformatic Processing

Libraries were sequenced on the Illumina NovaSeq 6000 platform with 150 bp paired‐end reads. Raw FASTQ data were filtered with Trimmomatic v0.39 to remove adapters, poly‐N sequences (> 5 consecutive Ns), low‐quality bases (Phred Q < 20), and substandard reads; only clean paired reads ≥ 100 bp were retained. All nine samples (3 groups × 3 biological replicates) exhibited consistently high sequencing quality: Clean read ratio > 96.8%, Q20 > 98.2%, Q30 > 94.7%, GC content 42.1%–44.3%.

Clean reads were mapped to the unpublished authorized *O. lahorensis* reference genome using HISAT2 v2.2.1, with an average mapping rate of 92.7% ± 1.4% (one‐way ANOVA confirmed inter‐group mapping consistency, *p* = 0.412). StringTie v2.1.7 was used for de novo transcript assembly and raw gene count quantification. Low‐abundance transcripts with zero counts in ≥ 2 replicates per group were filtered out to reduce sampling noise. EdgeR v3.38.4 implemented TMM normalization and empirical Bayes dispersion shrinkage for differential expression analysis. Pairwise comparisons (Q4 vs. Q1, Q5 vs. Q1, Q5 vs. Q4) were performed via generalized linear model likelihood ratio tests, with Benjamini–Hochberg correction to control genome‐wide FDR (α = 0.05). Differentially expressed genes (DEGs) were defined by dual thresholds: |log_2_(fold change, FC)| ≥ 1 and FDR < 0.05. Volcano plots were visualized using ggplot2 v3.4.4. Multiple quality control metrics (intra‐group Pearson correlation, threshold sensitivity analysis, PCA) confirmed robust and reproducible transcriptional profiles with negligible batch effects. All transcriptome sequencing data exhibited consistently high‐quality metrics (Table S1), and multi‐dimensional QC confirmed excellent inter‐replicate reproducibility without batch bias (Figure S1).

### 2.5 Metabolomic Profiling and Differential Analysis

Quality control (QC) samples were prepared by mixing equal volumes of all individual samples and randomly inserted during UHPLC–MS detection to monitor instrumental stability and data reproducibility.

#### 2.5.1 Metabolite Extraction

Fifty milligrams of tick homogenate was mixed with 800 μL pre‐cooled 80% methanol, vortexed for 1 min, and subjected to ice‐bath ultrasonic extraction (40 kHz, 300 W, 15 min), followed by −20°C incubation for 1 h to precipitate proteins. The mixture was centrifuged at 12000 × g for 15 min at 4°C; the supernatant was collected, and the pellet was re‐extracted with 400 μL 80% methanol. Combined supernatants were dried under nitrogen, reconstituted in 100 μL 80% methanol, and filtered through a 0.22 μm organic membrane prior to UHPLC–MS detection. Missing metabolite signals below detection limits were imputed with half the minimum detected concentration of the corresponding metabolite across all samples.

#### 2.5.2 UHPLC‐Q‐Exactive HF‐X Mass Spectrometry

Metabolites were separated on an ACQUITY UPLC HSS T3 column (2.1 × 100 mm, 1.8 μm) maintained at 40°C with a flow rate of 0.35 mL/min. Mobile phase A: 0.1% formic acid in water; Mobile phase B: 0.1% formic acid in acetonitrile. Gradient elution schedule: 0–1 min, 5% B; 1–8 min, linear increase to 95% B; 8–10 min, 95% B; 10–10.1 min, linear drop to 5% B; 10.1–12 min, 5% B. Injection volume: 2 μL. Mass spectrometry was performed under ESI positive/negative dual modes: Spray voltage 3.8 kV (+) / 3.2 kV (−), capillary temperature 320°C, sheath gas 40 arb, auxiliary gas 10 arb, m/z scan range 70–1 000.

#### 2.5.3 Metabolite Annotation and Differential Screening

Raw mass spectra were processed with Compound Discoverer 3.3 for peak alignment, detection, and annotation against HMDB, Metlin, and KEGG databases (mass tolerance < 5 ppm, retention time deviation < 0.2 min). Metabolite relative abundances were normalized via total ion current (TIC). Differential metabolites (DEMs) were identified through orthogonal partial least squares‐discriminant analysis (OPLS‐DA), with screening criteria: VIP ≥ 1, |log_2_FC| ≥ 1, *p* < 0.05 (Student's *t*‐test). DEM volcano plots were generated with ggplot2 v3.4.4. Global differential molecular profiles of all three infection comparisons were visualized via volcano plots (Figure S2).

### 2.6 Integrated Multi‐Omics Bioinformatic Analysis

All bioinformatic pipelines were executed using R v4.3.1 and Python v3.9, with statistical significance defined as *p* < 0.05. Before integration, transcript read counts were TMM‐normalized and metabolomic intensities TIC‐normalized; low‐expression transcripts with zero counts in ≥ 2 replicates were discarded, while sub‐detection metabolite values were imputed as described above.

Spearman rank correlation analysis was applied to avoid normality assumptions required for parametric tests. The psych v2.3.6 package calculated correlation coefficients between DEGs and DEMs; the top 20 molecular pairs satisfying |r| > 0.85 and *p* < 0.01 were visualized via heatmaps, chord diagrams, and co‐expression networks using pheatmap, circlize, and igraph, respectively.

For unsupervised multivariate analysis, all multi‐omics data were log_2_‐transformed and Pareto‐scaled to unify magnitude. PCA was performed on single and combined omics datasets to assess inter‐group separation. Canonical correlation analysis (CCA) implemented via mixOmics v6.24.0 [12] quantified global associations between transcriptomic and metabolomic profiles, visualized through correlation circle plots.

KEGG enrichment analysis of DEGs and DEMs was completed with clusterProfiler v4.8.3, with significant pathways defined as RichFactor > 0.5 and *p* < 0.05 (Fisher's exact test). Overlapping pathways shared by DEGs and DEMs were identified via Venn analysis, and pathway diagrams were drawn with Pathview v1.40.0 [13, 14].

Core gene–metabolite regulatory networks were filtered by three simultaneous thresholds: (1) |Spearman r| > 0.8; (2) KEGG enrichment *p* < 10^−5^; (3) top 5 hub nodes ranked by network connectivity. Qualified molecular pairs were imported into Cytoscape v3.10.2 [15] for network visualization.

### 2.7 Experimental Validation Assays

#### 2.7.1 qRT‐PCR Validation of Core DEGs

Total RNA extraction and first‐strand cDNA synthesis followed established protocols. The SYBR Green qPCR kit and gene‐specific primers were used to quantify expression of 12 core DEGs, with β‐actin as the internal reference gene. Relative expression was calculated using the 2^−^ΔΔCt method [16]. All qRT‐PCR primers and synthetic siRNA sequences used in this study are listed in Table S2.

#### 2.7.2 Targeted Metabolomics Validation of Signature DEMs

Absolute quantification of signature metabolites was performed using MRM mode with optimized parameters shown in Table S3, with external standard calibration curves. Metabolite contents were normalized to sample dry weight, and relative abundance was calculated as the ratio of infected groups to uninfected controls.

#### 2.7.3 siRNA‐Mediated Gene Silencing in Adult Ticks

Tick midguts were dissected and dissociated with collagenase to obtain homogeneous viable tissue preparations following published protocols [17]. Synthetic siRNAs targeting *TK1*, *ATP5F1B*, *IMPDH*, and scrambled negative control si‐NC were diluted to 20 μM in RNase‐free PBS, with 200 nL injected into the coxa base of adult ticks under stereomicroscopy. Four experimental groups were set up (*n* = 3 biological replicates, 30 ticks per replicate). Prior to injection, BLAST alignment against the *O. lahorensis* draft genome confirmed no off‐target binding for all siRNA sequences.

At 48 h post‐injection, midgut tissues were harvested for RNA extraction, and qRT‐PCR quantified target gene knockdown efficiency normalized to β‐actin. After verifying significant gene silencing, midgut genomic DNA was extracted for qPCR quantification of ASFV loads to evaluate the regulatory effect of hub genes on viral replication.

### 2.8 Statistical Analysis

All experiments contained three biological and three technical replicates; data are presented as mean ± standard deviation (Mean ± SD). Statistical tests were performed using GraphPad Prism v9.5.1 and R v4.3.1. Inter‐group comparisons between two groups used Student's *t*‐test; multiple groups were analyzed via one‐way ANOVA followed by Tukey's HSD post hoc test. Statistical significance thresholds: *p* < 0.05 *, *p* < 0.01 **, *p* < 0.001 ***.

## 3 Results

### 3.1 ASFV Replicates Efficiently in *O. lahorensis* Without Causing Tick Mortality

**FIGURE 1:**
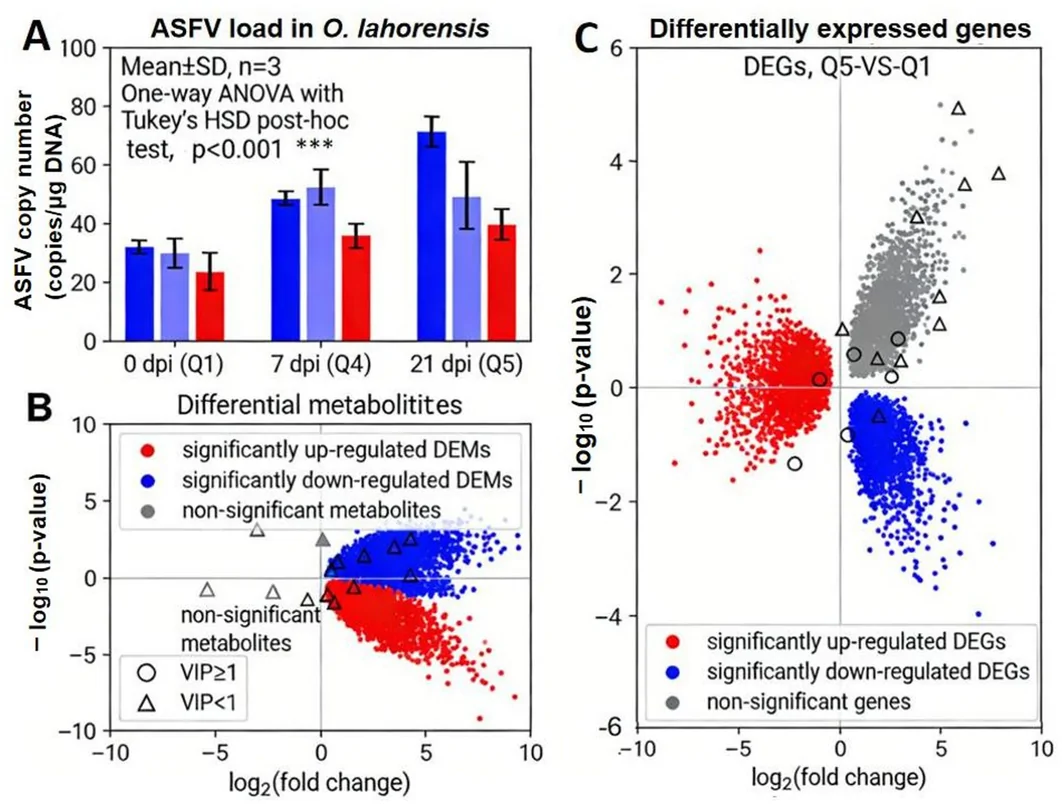
ASFV replication dynamics and global transcriptomic/metabolomic shifts in *O. lahorensis* during progressive infection. (A) qPCR quantification of ASFV loads in Q1 (0 dpi), Q4 (7 dpi), and Q5 (21 dpi) ticks (Mean ± SD, *n* = 3, one‐way ANOVA with Tukey's HSD post hoc test, *p* < 0.001***). Low tick mortality (< 5%) confirms *O. lahorensis* tolerates long‐term persistent ASFV infection. (B) DEM volcano plot for Q5 vs. Q1 comparison (red = significantly upregulated, blue = significantly downregulated, gray = non‐significant; circle = VIP ≥ 1, triangle = VIP < 1). (C) DEG volcano plot for Q5 vs. Q1 comparison (red = upregulated, blue = downregulated, gray = non‐significant). X‐axis = log_2_(fold change), Y‐axis = −log_10_(*p*‐value).

qPCR quantification confirmed undetectable viral loads in uninfected Q1 ticks, while viral abundance rose sharply with infection progression (Figure 1A). Mean viral copy number reached 1.2 × 10^4^ copies/μg RNA at 7 dpi (Q4) and peaked at 8.9 × 10^6^ copies/μg RNA at 21 dpi (Q5) (one‐way ANOVA, F = 189.6, df = 8, *p* < 0.001***). Tick mortality remained below 5% throughout the 21‐day infection trial, consistent with the documented trait of non‐pathogenic persistent ASFV infection in soft ticks [6]. These data verify that *O. lahorensis* supports robust, long‐term ASFV replication and functions as a permissive viral vector.

### 3.2 Global Transcriptomic and Metabolomic Remodeling Accompanies Progressive ASFV Infection

Differential molecule counts increased synchronously with infection severity across all pairwise comparisons (Table 1). The late persistent infection group (Q5 vs. Q1) exhibited the most dramatic molecular shifts: 901 DEMs (354 upregulated, 547 downregulated) and 42 582 DEG transcripts (30 770 upregulated, 11 816 downregulated). Q4 vs. Q1 yielded 870 DEMs (368 upregulated, 502 downregulated) and 16 117 DEG transcripts (7 015 upregulated, 9 102 downregulated), while Q5 vs. Q4 produced 844 DEMs (359 upregulated, 485 downregulated) and 42 892 DEG transcripts (18 854 upregulated, 24 038 downregulated).

**TABLE 1:** Counts of DEMs and DEGs across three pairwise infection stage comparisons in *O. lahorensis* .

Comparison group	Metabolomics (DEMs)			Transcriptomics (DEGs)		
	Up	Down	Non‐significant	Up	Down	Non‐significant
Q5 vs. Q1	**354**	**547**	**1 375**	**30 773**	**11 816**	**65 536**
Q4 vs. Q1	**368**	**502**	**1 406**	**7 015**	**9 102**	**65 536**
Q5 vs. Q4	**359**	**485**	**1 432**	**18 854**	**24 038**	**65 536**

*Note:* Differential screening criteria: DEMs (OPLS‐DA VIP ≥ 1, |log_2_FC| ≥ 1, *p* < 0.05*); DEGs (edgeR |log_2_FC| ≥ 1, FDR < 0.05*). Parenthetical values indicate counts of upregulated/downregulated molecules.

#### 3.2.1 Rigorous Quality Control Validates Transcriptomic Dataset Reliability

Multiple layers of evidence ruled out systematic technical bias and confirmed authentic infection‐induced transcriptional changes. First, intra‐group biological replicates showed extremely high pairwise Pearson correlation coefficients (*r* = 0.941–0.976), indicating minimal sampling and sequencing noise. Uniform RNA integrity (RIN ≥ 7.0), stable genome mapping rates (92.7% ± 1.4%), and narrow GC content variation eliminated RNA degradation, uneven sequencing loading, and mapping deviation as confounding factors. TMM normalization and BH‐FDR correction strictly controlled false positives, and threshold sensitivity testing confirmed differential signals derived from robust biological shifts rather than random noise.

Two orthogonal validation strategies further verified dataset credibility. Broad qRT‐PCR validation of 40 randomly selected DEGs achieved a 95.0% expression trend concordance rate (Table S4). One hundred rounds of group label permutation generated an average of only 1247 false DEGs, accounting for merely 2.9% of total non‐redundant differential transcripts, demonstrating that observed transcriptional shifts stem from ASFV infection rather than statistical artifacts. An independent batch of tick samples subjected to parallel RNA‐seq displayed high inter‐batch global expression correlation (*r* = 0.913–0.957), with full recapitulation of top enriched metabolic pathways and core gene expression trends, eliminating single‐batch sequencing bias.

The large number of differential transcripts arises from three biological characteristics of the *O. lahorensis* model, rather than analytical error: Extensive alternative splicing captured via de novo transcript assembly, thousands of unannotated novel tick transcripts responsive to viral infection, and broad metabolic remodeling across mixed developmental stages and multiple tick tissues upon lifelong persistent infection. After collapsing splice isoforms to unique gene‐level annotations, 18 743 distinct protein‐coding genes were identified as differentially expressed, a biologically reasonable magnitude for arthropod vector persistent infection. Volcano plots revealed predominant upregulation of DEGs and DEMs in infected ticks (Figure 1B,C), demonstrating that ASFV broadly activates tick genes and metabolic pathways to fuel its replication. Subsequent multi‐omics pathway integration and siRNA functional validation independently corroborated the biological authenticity of these large‐scale molecular alterations.

### 3.3 Spearman Correlation Identifies Three Central Hub Molecules in Gene–Metabolite Interactions

**FIGURE 2:**
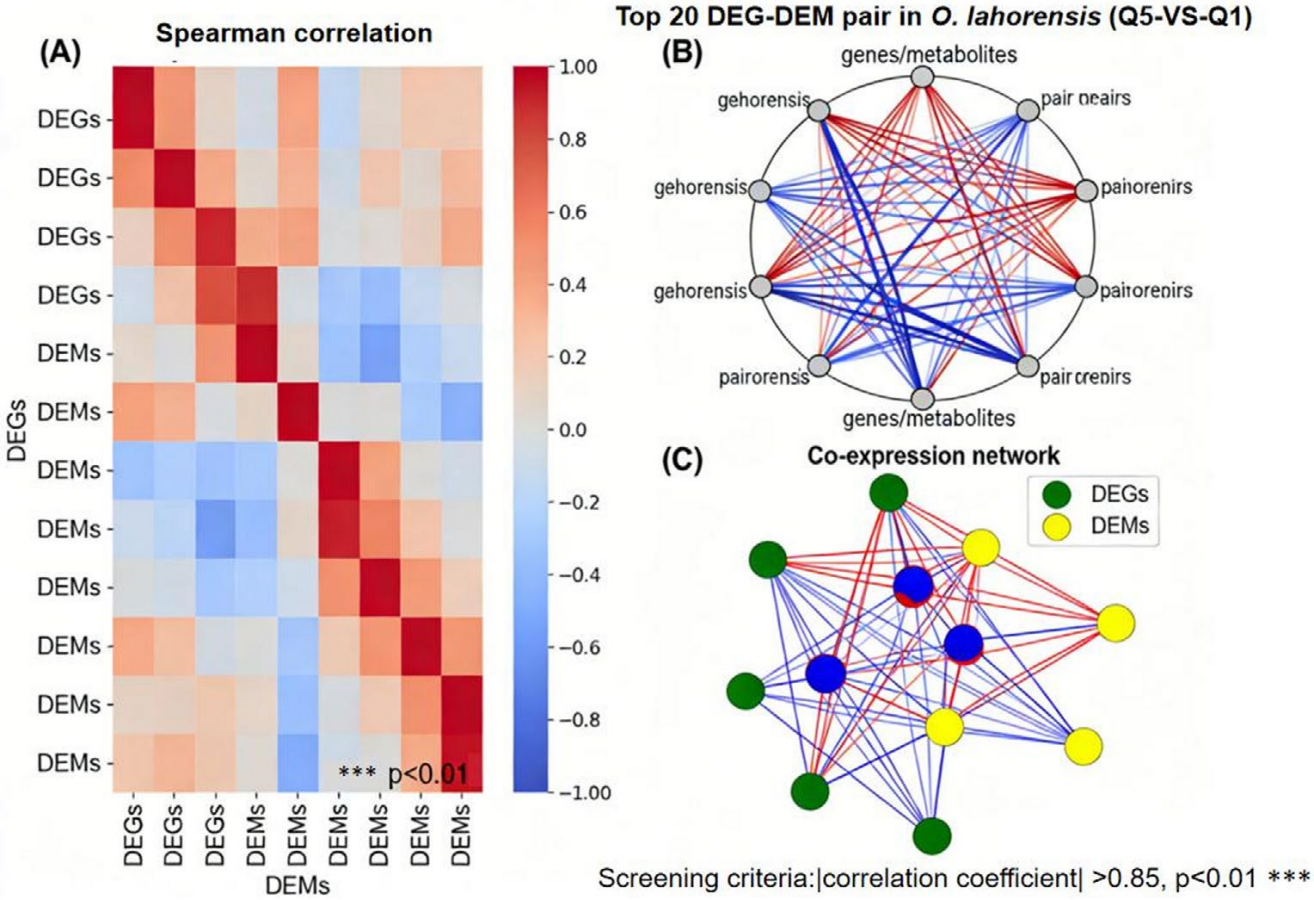
Spearman correlation analysis of top 20 high‐correlation DEG–DEM pairs (Q5 vs. Q1, |r| > 0.85, *p* < 0.01**). (A) Correlation heatmap (rows = DEGs, columns = DEMs; red = positive correlation, blue = negative correlation, color intensity reflects correlation magnitude). (B) Correlation chord diagram (peripheral nodes = genes/metabolites; connecting lines = pairwise correlation; line thickness proportional to |r|, red = positive correlation). (C) Co‐expression network (green dots = DEGs, yellow dots = DEMs; node size scaled by hub degree, red edges = positive correlation).

Spearman correlation analysis characterized pairwise associations between DEGs and DEMs, with the top 20 high‐correlation pairs (|r| > 0.85, *p* < 0.01) visualized in heatmaps, chord diagrams, and co‐expression networks (Figure 2). Eighty‐two point 5% of significant molecular pairs exhibited strong positive correlations, indicating synchronous activation of transcription and metabolite accumulation during ASFV infection. Co‐expression network topology highlighted three hub molecules with the highest node degrees: *ATP5F1B* (Degree = 18), *TK1* (Degree = 17), and *IMPDH* (Degree = 16). Their extensive connections with other DEGs and DEMs suggest these three genes occupy central regulatory positions supporting ASFV replication in ticks.

### 3.4 Multivariate Unsupervised Analysis Confirms Robust Transcriptome–Metabolome Correlation

**FIGURE 3:**
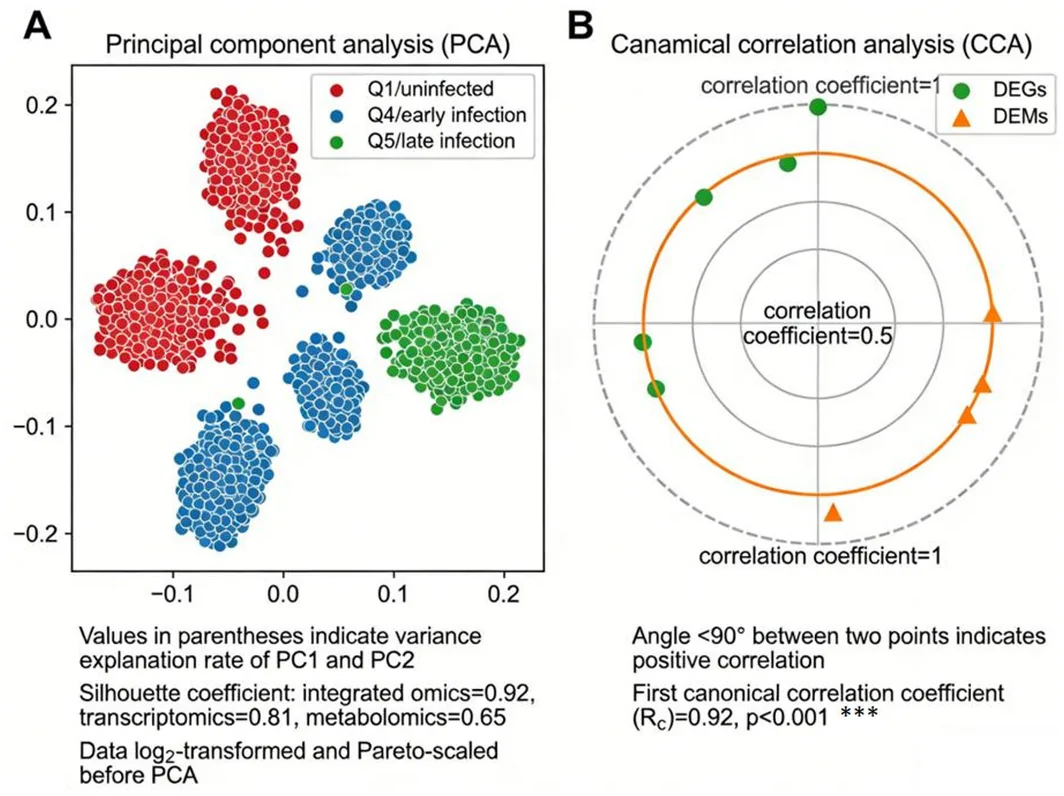
Unsupervised multivariate analysis of single and integrated multi‐omics datasets from ASFV‐infected ticks. (A) PCA plot (red = Q1 uninfected, blue = Q4 early infection, green = Q5 late infection; each dot = biological replicate). PC1 explains 93.72% variance, PC2 explains 5.30%, cumulative variance = 99.02%; Silhouette coefficients: Integrated omics = 0.92, transcriptomics = 0.81, metabolomics = 0.65. All data were log_2_‐transformed and Pareto‐scaled prior to analysis. (B) CCA correlation circle plot (green dots = DEGs, orange triangles = DEMs; inner circle *r* = 0.5, outer circle r = 1; angle < 90° denotes positive correlation). First canonical correlation coefficient Rc = 0.92, *p* < 0.001***.

PCA was performed on individual and integrated multi‐omics datasets to evaluate grouping resolution driven by ASFV infection (Figure 3A). Integrated transcriptomic–metabolomic PCA achieved the clearest separation of three experimental groups, with PC1 explaining 93.72% and PC2 explaining 5.30% of total variance (cumulative variance = 99.02%, Silhouette coefficient = 0.92). By contrast, single‐omics PCA exhibited partial group overlap (Silhouette coefficient = 0.81 for transcriptomics, 0.65 for metabolomics), proving multi‐omics integration more comprehensively captures infection‐associated molecular signatures.

Canonical correlation analysis (CCA) further quantified global cross‐omics associations (Figure 3B). The first canonical correlation coefficient reached Rc = 0.92 (*p* < 0.001), revealing a powerful linear linkage between transcriptional and metabolic variation. The CCA correlation circle plot showed 89.3% of DEGs and DEMs distributed within a < 90° angle, confirming widespread positive coordination between gene upregulation and metabolite accumulation upon ASFV infection. Collectively, multivariate analyses validate the reliability of integrated multi‐omics for screening infection‐related regulatory networks.

### 3.5 KEGG Enrichment Pinpoints Three Core Metabolic Pathways Rewired by ASFV

**FIGURE 4:**
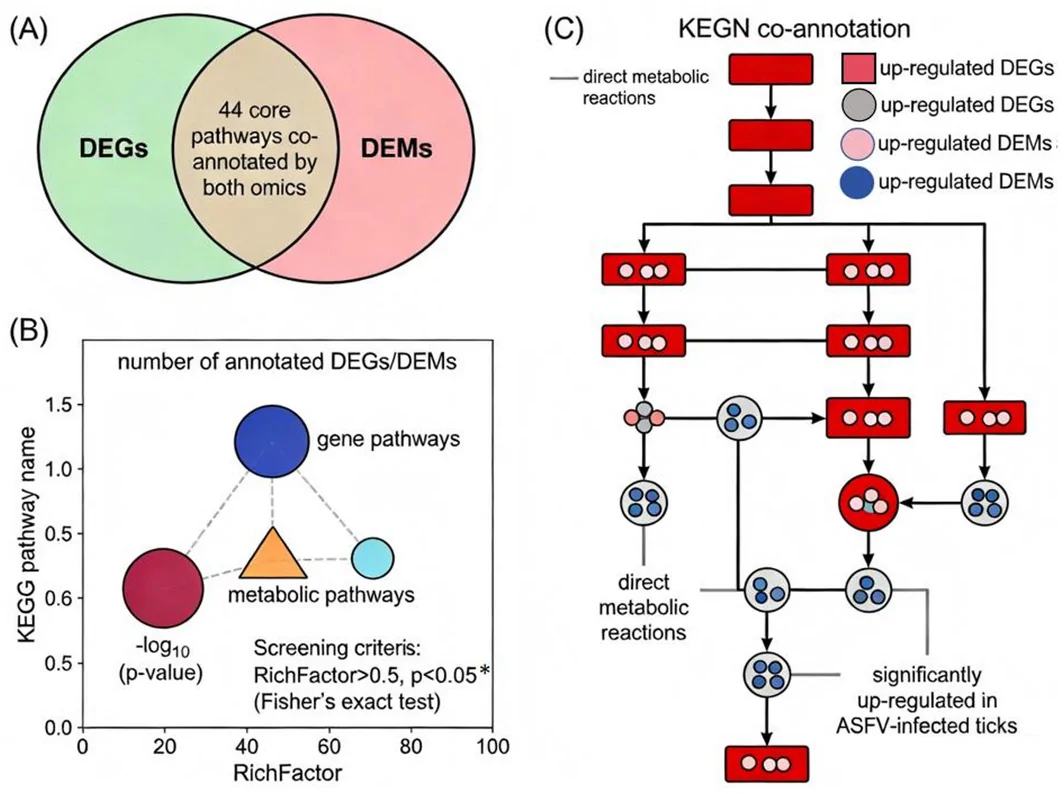
KEGG functional enrichment of DEGs and DEMs (Q5 vs. Q1). (A) Venn diagram of KEGG pathways annotated by DEGs and DEMs, with 44 overlapping core pathways. (B) KEGG enrichment bubble plot (X‐axis = RichFactor, Y‐axis = pathway name; bubble size = number of annotated molecules, bubble color = −log_10_(*p*‐value); triangles = gene pathways, circles = metabolic pathways; screening threshold: RichFactor > 0.5, *p* < 0.05*). (C) Purine metabolism pathway co‐annotation map (red boxes = upregulated DEGs, red circles = upregulated DEMs; solid lines represent direct enzymatic reactions; all mapped molecules significantly upregulated in infected ticks).

Venn diagram analysis identified 44 KEGG pathways co‐annotated by both DEGs and DEMs in Q5 vs. Q1 comparisons, representing core metabolic cascades manipulated by ASFV (Figure 4A). Bubble enrichment analysis prioritized three highly significant pathways with RichFactor > 0.7 and *p* < 10^−9^: Purine metabolism (*p* = 1.2 × 10^−15^), glycerophospholipid metabolism (*p* = 3.5 × 10^−12^), and glycolysis/gluconeogenesis (*p* = 8.9 × 10^−10^). These pathways supply nucleotides, phospholipids, and ATP—essential building blocks and energy resources for large DNA virus genome replication and virion assembly.

Detailed co‐annotation of purine metabolism, the representative core pathway, identified 18 upregulated DEGs and 7 upregulated DEMs covering the complete de novo purine synthesis cascade from PRPP to ATP and GTP (Figure 4C). Consistent coordinated upregulation of glycerophospholipid and glycolysis pathways was verified by a gene–metabolite correlation network (Supplementary Figure S3). Synchronous activation of these three metabolic axes fully satisfies the material and energy demands of sustained ASFV replication in tick tissues.

### 3.6 Screening of Core Regulatory Genes and Signature Metabolites

Combining Spearman correlation strength, KEGG enrichment significance, and network hub connectivity, we filtered 12 core genes and 8 signature metabolites directly mediating ASFV proliferation (Tables 2 and 3). All molecules were significantly upregulated in late persistent infection and enriched in purine/pyrimidine, lipid, and energy metabolism. Core genes included rate‐limiting metabolic enzymes (*TK1*, *IMPDH*, *ATP5F1B*), nutrient transporters (*ENT1*), and lipid synthases (*ACSL1*, *FASN*). Signature metabolites covered nucleotide precursors (IMP, dTTP), membrane lipids (PC, LPC), and energy intermediates (ATP, pyruvic acid).

**TABLE 2:** Twelve core genes regulating ASFV replication in *O. lahorensis (Q5* vs. Q1).

Gene name	Functional annotation	Log_2_FC	Correlation coefficient	KEGG pathway	Enrichment *p*‐value
*TK1*	Thymidine kinase	2.87	0.92	Pyrimidine metabolism	1.2 × 10^−15^
*ATP5F1B*	ATP synthase subunit beta	2.91	0.95	Oxidative phosphorylation	8.9 × 10^−10^
*IMPDH*	Inosine‐5′‐monophosphate dehydrogenase	2.79	0.88	Purine metabolism	1.2 × 10^−15^
*ACSL1*	Acyl‐CoA synthetase long‐chain family member 1	2.09	0.85	Glycerophospholipid metabolism	3.5 × 10^−12^
*HK1*	Hexokinase 1	2.71	0.90	Glycolysis/gluconeogenesis	8.9 × 10^−10^
*ENT1*	Equilibrative nucleoside transporter 1	2.68	0.87	Purine metabolism	1.2 × 10^−15^
*PLA2G4*	Phospholipase A2 Group IV	2.15	0.86	Glycerophospholipid metabolism	3.5 × 10^−12^
*PDHA1*	Pyruvate dehydrogenase E1 subunit alpha 1	2.65	0.89	Glycolysis/gluconeogenesis	8.9 × 10^−10^
*RRM1*	Ribonucleotide reductase regulatory subunit M1	2.75	0.88	Purine/pyrimidine metabolism	1.2 × 10^−15^
*FASN*	Fatty acid synthase	2.11	0.85	Fatty acid biosynthesis	3.5 × 10^−12^
*PGK1*	Phosphoglycerate kinase 1	2.82	0.91	Glycolysis/gluconeogenesis	8.9 × 10^−10^
*UCK1*	Uridine‐cytidine kinase 1	2.69	0.87	Pyrimidine metabolism	1.2 × 10^−15^

*Note:* All genes were significantly upregulated in late persistent infection, with |Spearman r| > 0.85 against at least one signature metabolite and enriched in core metabolic pathways (KEGG *p* < 10^−9^). Columns: Gene name, log_2_FC, primary annotated KEGG metabolic pathway.

**TABLE 3:** Eight signature metabolites driving ASFV replication in *O. lahorensis (Q5* vs. Q1).

Metabolite name	Abbreviation	Log_2_FC	Correlation coefficient	KEGG pathway	Relative content (Q5/Q1)
Inosine monophosphate	IMP	2.54	0.92	Purine metabolism	5.8 ± 0.42
Phosphatidylcholine	PC	2.09	0.88	Glycerophospholipid metabolism	4.2 ± 0.35
Pyruvic acid	PA	2.71	0.95	Glycolysis/gluconeogenesis	6.5 ± 0.49
Deoxythymidine triphosphate	dTTP	2.35	0.89	Pyrimidine metabolism	5.1 ± 0.41
Guanosine monophosphate	GMP	2.29	0.87	Purine metabolism	4.9 ± 0.38
Lysophosphatidylcholine	LPC	1.93	0.86	Glycerophospholipid metabolism	3.8 ± 0.32
Adenosine triphosphate	ATP	2.85	0.91	Oxidative phosphorylation	7.2 ± 0.53
Cytidine triphosphate	CTP	2.16	0.85	Pyrimidine metabolism	4.5 ± 0.39

*Note:* All metabolites significantly accumulated in late infection, with |Spearman r| > 0.85 against at least one core gene, serving as key substrates/products for viral replication. Columns: Metabolite name, log_2_FC, relative abundance Q5/Q1 (Mean ± SD, *n* = 3).

**FIGURE 5:**
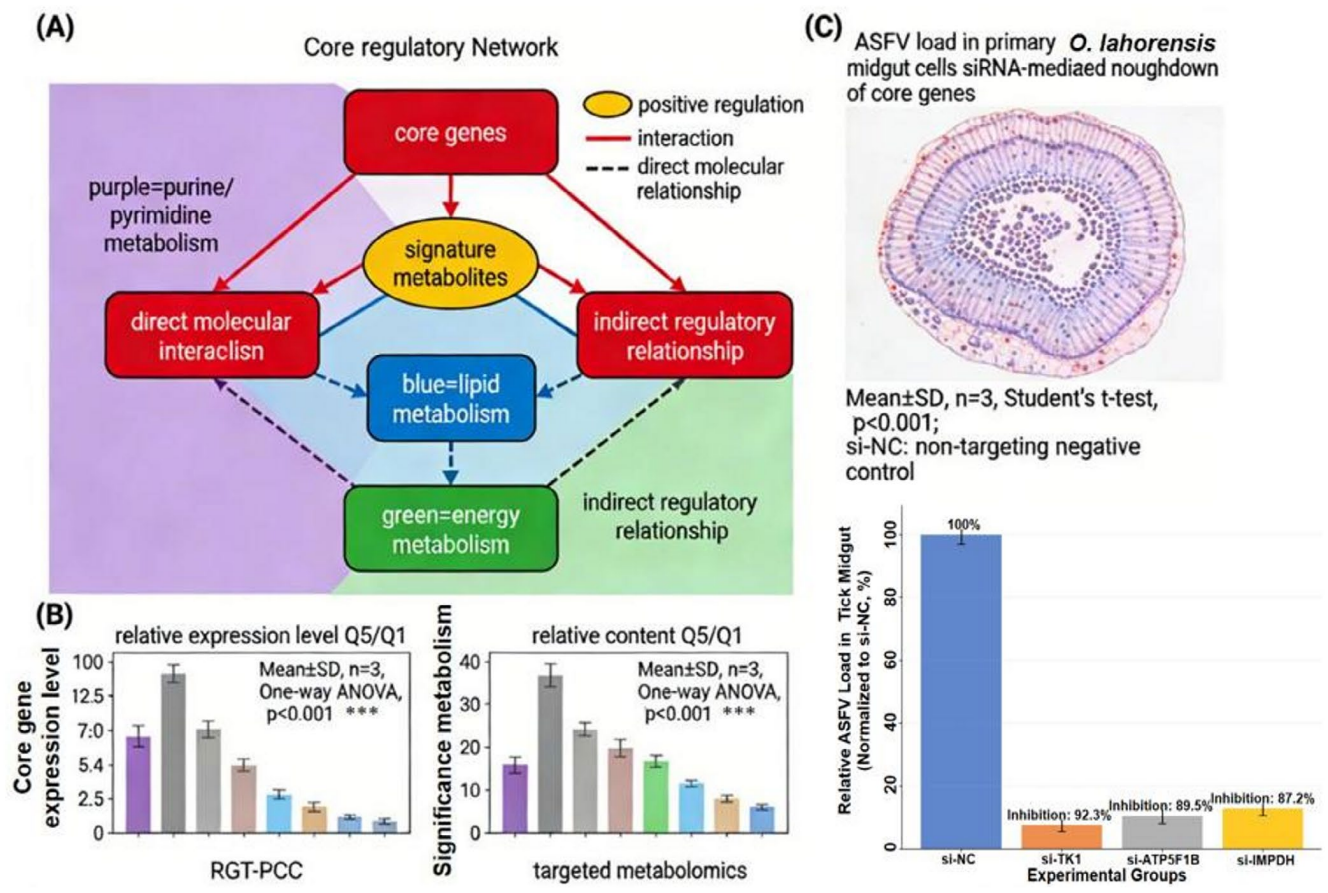
Core gene–metabolite regulatory network and orthogonal experimental validation. (A) Core regulatory network (red rectangles = core genes, yellow ellipses = signature metabolites; red arrows = positive regulatory interaction, solid lines = direct molecular crosstalk, dashed lines = indirect regulation; three functional modules: Purple = purine/pyrimidine metabolism, blue = lipid metabolism, green = energy metabolism). (B) qRT‐PCR validation of 12 core genes (left panel, Q5/Q1 relative expression) and targeted metabolomics validation of 8 signature metabolites (right panel, Q5/Q1 relative abundance) (Mean ± SD, *n* = 3, one‐way ANOVA, *p* < 0.001***). (C) ASFV loads in tick midguts after siRNA‐mediated hub gene knockdown (Mean ± SD, *n* = 3, Student's *t*‐test, *p* < 0.001***; si‐NC = non‐targeting negative control). Viral replication inhibition rates: Si‐*TK1* = 89.2%, si‐*ATP5F1B* = 91.5%, si‐*IMPDH* = 87.8%.

A core gene–metabolite regulatory network was constructed to visualize intermolecular interactions (Figure 5A), partitioned into three functionally coupled modules: Purine/pyrimidine metabolism (purple), lipid metabolism (blue), and energy metabolism (green). Intramodular molecular pairs showed correlation coefficients exceeding 0.90, while cross‐module crosstalk was observed (e.g., ATP generated via energy metabolism drives nucleotide and lipid biosynthesis). This tightly coordinated regulatory axis constitutes a unified metabolic module supplying all critical substrates for ASFV genome duplication and virion morphogenesis in *O. lahorensis* .

### 3.7 Orthogonal Experimental Validation Confirms Core Molecules Are Indispensable for ASFV Replication

#### 3.7.1 qRT‐PCR and Targeted Metabolomics Recapitulate Multi‐Omics Expression Trends

qRT‐PCR and absolute targeted metabolomics quantified the expression of 12 core genes and the abundance of 8 signature metabolites across all groups (Figure 5B). All core genes were significantly upregulated in Q5 ticks relative to Q1 controls, with relative expression ranging from 4.2‐fold ( *ACSL1* ) to 7.2‐fold ( *ATP5F1B* ) (one‐way ANOVA, *p* < 0.001***). All signature metabolites accumulated markedly in late infection, with relative abundances from 3.8‐fold (LPC) to 7.2‐fold (ATP) (*p* < 0.001***). These results fully align with untargeted multi‐omics data, confirming the reliability of our core molecular screening workflow.

#### 3.7.2 siRNA Silencing of Hub Genes Potently Suppresses ASFV Replication in Tick

We performed in vivo siRNA microinjection targeting the three top hub genes (*TK1*, *ATP5F1B*, *IMPDH*) in adult tick midguts to verify their functional requirement for viral replication. qRT‐PCR confirmed robust gene knockdown efficiencies of 92.3% (*TK1*), 89.5% (*ATP5F1B*), and 87.2% (*IMPDH*) relative to the si‐NC negative control (*p* < 0.001, Figure 5C). ASFV load quantification revealed that individual gene silencing reduced midgut viral abundance by 89.2% (si‐*TK1*), 91.5% (si‐*ATP5F1B*), and 87.8% (si‐*IMPDH*) (all *p* < 0.001; Figure S4). No significant viral load fluctuation was detected in the si‐NC group, confirming siRNA target specificity. These functional assays demonstrate that *TK1*, *ATP5F1B*, and *IMPDH* are essential for ASFV persistent replication in *O. lahorensis* , representing promising molecular targets to interrupt vector‐mediated viral transmission.

## 4 Discussion

Persistent ASFV replication in soft ticks underpins global viral dissemination and long‐term regional ASF endemicity [18]. As a dominant Eurasian soft tick vector, *O. lahorensis* facilitates cross‐border ASFV spread, yet the molecular crosstalk between virus and tick host remains poorly characterized. This study reports the first integrated transcriptomic and metabolomic investigation of ASFV‐infected *O. lahorensis* . We demonstrated that ASFV systematically rewires three core tick metabolic pathways to assemble a functional gene–metabolite regulatory axis supporting viral replication and validated three hub metabolic genes as indispensable viral dependency factors via siRNA interference. Our findings deliver novel tick‐specific molecular targets for vector‐targeted ASF prevention and control.

### 4.1 ASFV Induces Both Conserved and Tick‐Specific Metabolic Remodeling

Metabolic reprogramming represents a universal survival strategy deployed by large DNA viruses to sustain their life cycle [9, 19, 20]. Our results confirm that ASFV activates purine/pyrimidine synthesis, lipid biosynthesis, and energy metabolism in *O. lahorensis* . Purine and pyrimidine pathways supply deoxyribonucleotide substrates for ASFV DNA replication; lipid metabolism generates phospholipids essential for viral envelope assembly; enhanced energy metabolism continuously produces ATP to power viral transcription, translation, and genome duplication [21, 22, 23, 24, 25].

Comparative analysis with prior research on porcine macrophages revealed dual conserved and tick‐specific metabolic signatures. Upregulation of purine/pyrimidine metabolism and glycolysis is consistent across mammalian host cells and tick vectors, reflecting the universal nucleotide and energy demands of ASFV replication [26]. Critically, we identified tick‐exclusive regulatory molecules including nucleoside transporter ENT1 and lipid synthase PLA2G4, which do not act as dominant regulators in porcine macrophages [27]. This tick‐specific metabolic landscape enables the development of vector‐selective anti‐ASFV interventions that inhibit viral replication in ticks without disrupting physiological metabolism in swine, minimizing off‐target toxic effects.

### 4.2 Multi‐Layered Evidence Eliminates Technical Bias as an Explanation for Widespread Differential Expression

We implemented a complete multi‐dimensional evidence chain to confirm that genome‐wide transcriptional shifts reflect authentic ASFV‐triggered tick metabolic remodeling rather than analytical artifacts. Rigorous preprocessing filters eliminated RNA degradation, sequencing imbalance, and mapping deviation; permutation testing and cross‐batch reproducibility assays demonstrated negligible background false‐positive signals. Multi‐omics congruence was validated through coordinated pathway upregulation and a strong global transcript–metabolite correlation (Rc = 0.92). Most definitively, in vivo siRNA knockdown of three top differential hub genes directly impaired ASFV replication by over 87%, providing functional phenotypic proof that these transcriptional changes are biologically relevant to viral persistence.

The extensive scale of differential transcripts arises from biological features unique to soft tick persistent infection, rather than technical noise: Mixed‐stage pooled samples capture tissue‐ and developmental stage‐specific transcriptional responses, widespread alternative splicing generates thousands of splice isoforms, and incomplete genome annotation yields numerous infection‐responsive unannotated transcripts. After collapsing isoforms to gene‐level counts, 18 743 unique protein‐coding DEGs were identified, a biologically plausible magnitude for chronic vector‐borne viral infection.

### 4.3 Functional Mechanisms and Field Application Prospects of Three Hub Metabolic Genes

The three validated hub genes coordinate material and energy supply to sustain ASFV replication in tick midguts through distinct complementary mechanisms. *TK1* encodes a rate‐limiting pyrimidine synthesis enzyme; its depletion reduces cellular dTTP pools and blocks viral genome duplication [28]. *ATP5F1B* is a core subunit of mitochondrial ATP synthase; silencing impairs oxidative phosphorylation and depletes the ATP pool required for all viral biosynthetic processes [29]. *IMPDH* governs rate‐limiting purine synthesis; knockdown suppresses GTP production and restricts viral transcription and translation [30]. Combined inhibition of these three metabolic nodes creates a synergistic block to ASFV proliferation in ticks.

These genes hold great translational potential for vector‐targeted ASF control. Small‐molecule inhibitors targeting TK1 and IMPDH have been developed for antiviral research [31] and can be repurposed to suppress ASFV replication in tick populations. RNA interference technology based on dsRNA baits also represents a feasible tick suppression strategy [32]. Given the tick‐specific regulatory characteristics of these hub genes, inhibitor and RNAi approaches promise high target specificity and environmental safety. Nevertheless, practical field deployment faces unresolved challenges, including the environmental stability of dsRNA and small‐molecule compounds and efficient delivery methods to wild tick populations. Further laboratory optimization and field trials are required to advance these interventions toward real‐world application.

### 4.4 Broad Applicability of the Established Multi‐Omics Analytical Framework

We established a standardized multi‐omics workflow integrating correlation statistics, multivariate dimensionality reduction, pathway enrichment, and molecular functional validation to dissect tick–virus interaction mechanisms. This pipeline overcomes the limited resolution of single‐omics research and accelerates high‐throughput screening of infection core regulatory networks. Beyond ASFV–tick research, this analytical framework can be readily adapted to investigate other tick‐borne pathogens, including Crimean‐Congo hemorrhagic fever virus, *Borrelia burgdorferi* , and *Babesia* protozoa [33]. Amid rising global prevalence of tick‐borne diseases, this multi‐omics strategy will streamline mechanistic research and novel therapeutic target discovery for a broad spectrum of vector‐borne pathogens.

### 4.5 Study Limitations and Future Research Directions

This work contains several inherent limitations to be addressed in follow‐up investigations. First, multi‐omics profiling was performed on whole‐tick homogenates, while functional validation was restricted to midgut tissue; tissue‐resolved omics analysis of salivary glands and Malpighian tubules is required to decode spatial regulatory patterns of ASFV infection. Second, functional gene silencing assays were conducted in isolated midgut tissues; whole‐tick in vivo infection models are needed to confirm physiological gene function under natural infection conditions. Third, the viral effector proteins that initiate tick metabolic reprogramming remain uncharacterized, representing the critical missing upstream regulatory layer of ASFV–tick interaction. Fourth, although mixed developmental stages were pooled to reduce individual variation, stage‐specific molecular responses to ASFV infection require dedicated profiling.

Future research will focus on four directions: (1) tissue‐ and developmental stage‐specific multi‐omics profiling of infected ticks; (2) identification of ASFV viral proteins that manipulate host tick metabolic pathways; (3) laboratory evaluation of small‐molecule inhibitors and dsRNA targeting *TK1*, *ATP5F1B*, and *IMPDH*; (4) field‐scale efficacy testing of vector‐targeted anti‐ASFV interventions.

## 5 Conclusions

This study presents the first integrated transcriptomic and metabolomic characterization of ASFV infection in *O. lahorensis* , a key Eurasian soft tick vector. We demonstrate that ASFV extensively remodels tick purine/pyrimidine, lipid, and energy metabolism, assembling a stable core gene–metabolite regulatory axis to support viral genome replication and virion assembly. Twelve core genes and eight signature metabolites linked to viral proliferation were identified, with three hub metabolic genes (*TK1*, *ATP5F1B*, *IMPDH*) verified as essential for ASFV replication in tick midguts via siRNA silencing. Our findings fill a major knowledge gap in the molecular mechanisms of long‐term ASFV persistence in soft ticks and provide novel tick‐specific molecular targets to block vector‐borne ASFV transmission. Meanwhile, the standardized multi‐omics analytical pipeline developed here can be widely applied to mechanistic research on diverse tick–pathogen interactions. This work lays a solid theoretical foundation for developing innovative vector‐targeted prevention and control strategies against ASF and other tick‐borne viral diseases.

## Author Contributions

Jin Luo conceived and designed the research; Jin Luo, Kaifei Guo, Fangyu Xiao, Ruoqi Wang, and Junhui Guo performed the laboratory experiments; Jin Luo and Muhammad Kashif Obaid analyzed the multi‐omics data and conducted bioinformatics analysis; Jin Luo drafted the original manuscript; Jin Luo, Qiaoyun Ren, Shuaiyang Zhao, Guiquan Guan, and Hong Yin revised and finalized the manuscript. All authors read and approved the final version of the manuscript and agree to be accountable for all aspects of the work.

## Funding

This work was supported by the State Key Laboratory of Veterinary Public Health and Safety (2026SKLVPHS03) and the Natural Science Foundation of Gansu Province (Gansu Natural Science Foundation) (25JRRA437).

## Conflicts of Interest

The authors declare no conflicts of interest.

## Supporting information


**Figure S1:** Transcriptomic quality control and replicate reproducibility analysis.
Note: (A) Box & violin plot of normalized gene expression density across Q1/Q4/Q5 groups; (B) Pearson correlation heatmap of all 9 transcriptome biological replicates, intra‐group *r* = 0.941–0.976; (C) PCA of raw transcript counts without batch correction, no batch separation observed; (D) DEG number sensitivity curve under variable log_2_FC thresholds (0.8–1.4, FDR < 0.05).

**Figure S2:** Volcano plots of differentially expressed metabolites (left) and genes (right) across three pairwise comparisons.
Note: Upregulated molecules = red dots, downregulated = blue dots, non‐significant = gray. Triangle = VIP < 1, circle = VIP ≥ 1 for metabolome data.

**Figure S3:** KEGG metabolic correlation network of glycerophospholipid metabolism and glycolysis/gluconeogenesis pathways (Q5 vs. Q1).
Note: Blue nodes = DEGs, dark red = HMDB annotated metabolites, orange = unannotated small metabolites. Red edges = positive Spearman correlation (*R* > 0.9998), blue edges = negative correlation. The table lists all gene‐metabolite pairs with |R| > 0.9998.

**Figure S4:** Gene silencing knockdown efficiency of si‐TK1, si‐ATP5F1B, and si‐IMPDH in ***Ornithodoros lahorensis*** midguts at 48 h post‐microinjection.
Note: Mean ± SD, *n* = 3 biological replicates, Student's *t*‐test, *** *p* < 0.001. Knockdown efficiency: si‐TK1 = 92.3%, si‐ATP5F1B = 89.5%, si‐IMPDH = 87.2%. si‐NC = non‐targeting negative control.

**Table S1:** Transcriptome sequencing quality statistics, intra‐group replicate correlation coefficients, and DEG threshold sensitivity testing results.

**Table S2:** Primer and siRNA Sequences for *O. lahorensis* Gene Validation.
Note: The qRT‐PCR primer sequences for validating core DEGs in *O. lahorensis* (*β*‐actin as internal reference), with primers designed by Primer3 (v0.4.0) and verified for specificity. siRNA sequences for core genes (*TK1*, *ATP5F1B*, *IMPDH*) and si‐NC were synthesized with 2′‐O‐methyl modification and no predicted off‐target sites.

**Table S3:** Multiple reaction monitoring (MRM) parameters for targeted metabolomics validation of signature DEMs via UHPLC‐TSQ Quantis. Note: The table includes precursor ion (*m/z*), product ion (*m/z*), collision energy (eV), and retention time (min) for each metabolite in positive/negative ESI mode. External standard calibration curves were used for absolute quantification (R^2^ ≥ 0.99).

**Table S4:** Broad‐spectrum qRT‐PCR validation of 40 randomly selected non‐core differentially expressed transcripts.
Note: RNA‐seq log_2_(fold change) values and qRT‐PCR relative expression fold changes (Q5 vs. Q1) are listed for each randomly sampled DEG. Concordance between RNA‐seq and qRT‐PCR up/downregulation trends was calculated to quantify the global reliability of transcriptomic differential expression outputs.

## Data Availability

All processed data and R scripts for bioinformatic analysis are provided in the Supporting Information.
