# Supplementary material for: Transcriptomic and Metabolomic Profiling Identifies a Core Gene–Metabolite Axis Driving African Swine Fever Virus Replication in the Soft Tick Ornithodoros lahorensis
Source: FASEB J. 2026 Aug 31;40(17):e72256. doi: 10.1096/fj.202602001RR (PMC13528556; doi:10.1096/fj.202602001RR)
Supplement: Supplementary file 1 [file FSB2-40-e72256-s001.docx]

| **Sample ID** | **Group** | **Total Raw Reads (M)** | **Clean Read Ratio (%)** | **Q20 (%)** | **Q30 (%)** | **GC Content (%)** |
| --- | --- | --- | --- | --- | --- | --- |
| Q1-1 | Q1 | 7.8 | 97.2 | 98.6 | 95.1 | 43.2 |
| Q1-2 | Q1 | 8.1 | 97.0 | 98.5 | 94.9 | 43.1 |
| Q1-3 | Q1 | 7.9 | 97.3 | 98.7 | 95.2 | 43.3 |
| Q4-1 | Q4 | 8.2 | 96.9 | 98.4 | 94.8 | 42.9 |
| Q4-2 | Q4 | 7.7 | 97.1 | 98.3 | 95.0 | 43.0 |
| Q4-3 | Q4 | 8.0 | 97.2 | 98.6 | 95.3 | 42.8 |
| Q5-1 | Q5 | 7.6 | 97.4 | 98.8 | 95.4 | 43.5 |
| Q5-2 | Q5 | 7.9 | 97.0 | 98.5 | 95.0 | 43.4 |
| Q5-3 | Q5 | 8.3 | 97.1 | 98.6 | 95.2 | 43.6 |

**Supplementary Table S1. Transcriptome sequencing quality statistics, intra-group replicate correlation coefficients, and DEG threshold sensitivity testing results.**
