# Supplementary material for: Transcriptomic and Metabolomic Profiling Identifies a Core Gene–Metabolite Axis Driving African Swine Fever Virus Replication in the Soft Tick Ornithodoros lahorensis
Source: FASEB J. 2026 Aug 31;40(17):e72256. doi: 10.1096/fj.202602001RR (PMC13528556; doi:10.1096/fj.202602001RR)
Supplement: Supplementary file 2 [file FSB2-40-e72256-s002.docx]

**Supplementary Figure S3.** **KEGG metabolic correlation network of glycerophospholipid metabolism and glycolysis/gluconeogenesis pathways (Q5 vs Q1)**.****

**Note:** Blue nodes = DEGs, dark red = HMDB annotated metabolites, orange = unannotated small metabolites. Red edges = positive Spearman correlation (R>0.9998), blue edges = negative correlation. Table lists all gene-metabolite pairs with |R|>0.9998.

| **Gene** | **Metab** | **R** |
| --- | --- | --- |
| TRINITY_DN3533_c0_g1_i8-Q5_2 | HMDB0242392 | 0.999883812130419 |
| TRINITY_DN3533_c0_g1_i8-Q5_2 | 3.514_279.13398 | -0.999876073 |
| TRINITY_DN650_c0_g1_i1-Q5_1 | HMDB0242392 | 0.999870950404839 |
| TRINITY_DN3533_c0_g1_i8-Q5_2 | HMDB0000064 | 0.999867369613446 |
| TRINITY_DN3533_c0_g1_i8-Q5_2 | 5.02_487.23071 | 0.999860012323335 |
| TRINITY_DN650_c0_g1_i1-Q5_1 | 4.013_309.10826 | -0.999855973 |
| TRINITY_DN650_c0_g1_i1-Q5_1 | HMDB0000064 | 0.999849169810291 |
| TRINITY_DN3256_c0_g1_i3-Q5_1 | 3.514_279.13398 | -0.99984676 |
| TRINITY_DN650_c0_g1_i1-Q5_1 | 5.02_487.23071 | 0.999844958962737 |
| TRINITY_DN650_c0_g1_i1-Q5_1 | 3.514_279.13398 | -0.999840042 |
| TRINITY_DN3533_c0_g1_i8-Q5_2 | 4.013_309.10826 | -0.999835645 |
| TRINITY_DN17623_c0_g1_i1-Q5_3 | 4.013_309.10826 | -0.99983368 |
| TRINITY_DN21709_c0_g1_i3-Q5_2 | HMDB0242392 | 0.999832630585089 |
| TRINITY_DN3533_c0_g1_i8-Q5_2 | HMDB0000391 | 0.999832464075617 |
| TRINITY_DN6754_c0_g1_i2-Q1_3 | 1.107_318.11974 | -0.999830303 |
| TRINITY_DN3256_c0_g1_i3-Q5_1 | HMDB0242392 | 0.999829215366382 |
| TRINITY_DN44514_c0_g1_i1-Q5_3 | 3.514_279.13398 | -0.999827137 |
| TRINITY_DN6754_c0_g1_i2-Q1_3 | 9.322_411.25081 | 0.999821976688913 |
| TRINITY_DN17623_c0_g1_i1-Q5_3 | HMDB0242392 | 0.999820868325285 |
| TRINITY_DN3256_c0_g1_i3-Q5_1 | HMDB0000064 | 0.999818598831713 |
