# Supplementary material for: Transcriptomic and Metabolomic Profiling Identifies a Core Gene–Metabolite Axis Driving African Swine Fever Virus Replication in the Soft Tick Ornithodoros lahorensis
Source: FASEB J. 2026 Aug 31;40(17):e72256. doi: 10.1096/fj.202602001RR (PMC13528556; doi:10.1096/fj.202602001RR)
Supplement: Supplementary file 3 [file FSB2-40-e72256-s003.docx]

****Supplementary Table S3.**** **Multiple reaction monitoring (MRM) parameters for targeted metabolomics validation of signature DEMs via UHPLC-TSQ Quantis.** Note: The table includes precursor ion (m/z), product ion (m/z), collision energy (eV), and retention time (min) for each metabolite in positive/negative ESI mode. External standard calibration curves were used for absolute quantification (R²≥0.99).

| **Metabolite name** | **ESI mode** | **Precursor ion**  **(m/z)** | **Product ion**  **(m/z)** | **Collision energy**  **(eV)** | **Retention time**  **(min)** |
| --- | --- | --- | --- | --- | --- |
| ATP (Adenosine triphosphate) | Positive | 508.1 | 136.1 | 35 | 2.85 |
| ADP (Adenosine diphosphate) | Positive | 428.1 | 136.1 | 32 | 2.72 |
| AMP (Adenosine monophosphate) | Positive | 348.1 | 136.1 | 28 | 2.60 |
| GTP (Guanosine triphosphate) | Positive | 524.1 | 152.1 | 36 | 2.90 |
| Glucose-6-phosphate | Negative | 259.0 | 97.0 | 18 | 3.15 |
| Pyruvic acid | Negative | 87.0 | 43.0 | 12 | 1.85 |
| Lactic acid | Negative | 89.0 | 45.0 | 10 | 1.92 |
| Phosphatidylcholine (16:0/18:1) | Positive | 760.6 | 184.1 | 30 | 8.75 |
