# Supplementary material for: Transcriptomic and Metabolomic Profiling Identifies a Core Gene–Metabolite Axis Driving African Swine Fever Virus Replication in the Soft Tick Ornithodoros lahorensis
Source: FASEB J. 2026 Aug 31;40(17):e72256. doi: 10.1096/fj.202602001RR (PMC13528556; doi:10.1096/fj.202602001RR)
Supplement: Supplementary file 4 [file FSB2-40-e72256-s004.docx]

**Supplementary Figure S1. Transcriptomic quality control and replicate reproducibility analysis.**

**Note: Box & violin plot of normalized gene expression density across Q1/Q4/Q5 groups; (B) Pearson correlation heatmap of all 9 transcriptome biological replicates, intra-group r=0.941–0.976; (C) PCA of raw transcript counts without batch correction, no batch separation observed; (D) DEG number sensitivity curve under variable log₂FC thresholds (0.8–1.4, FDR<0.05).**
