# Supplementary material for: Transcriptomic and Metabolomic Profiling Identifies a Core Gene–Metabolite Axis Driving African Swine Fever Virus Replication in the Soft Tick Ornithodoros lahorensis
Source: FASEB J. 2026 Aug 31;40(17):e72256. doi: 10.1096/fj.202602001RR (PMC13528556; doi:10.1096/fj.202602001RR)
Supplement: Supplementary file 5 [file FSB2-40-e72256-s005.docx]

****Supplementary Figure S4.** Gene silencing knockdown efficiency of si-TK1, si-ATP5F1B and si-IMPDH in Ornithodoros lahorensis midguts at 48 h post microinjection.**

- **Note: Mean ± SD, n=3 biological replicates, Student’s t-test, *** p<0.001. Knockdown efficiency: si-TK1=92.3%, si-ATP5F1B=89.5%, si-IMPDH=87.2%. si-NC = non-targeting negative control.**
