# Supplementary material for: Transcriptomic and Metabolomic Profiling Identifies a Core Gene–Metabolite Axis Driving African Swine Fever Virus Replication in the Soft Tick Ornithodoros lahorensis
Source: FASEB J. 2026 Aug 31;40(17):e72256. doi: 10.1096/fj.202602001RR (PMC13528556; doi:10.1096/fj.202602001RR)
Supplement: Supplementary file 6 [file FSB2-40-e72256-s006.docx]

****Supplementary Table S2.**** **Primer and siRNA Sequences for *O. lahorensis* Gene Validation.**

Note: The qRT-PCR primer sequences for validating core DEGs in *O. lahorensis* (*β*-actin as internal reference), with primers designed by Primer3 (v0.4.0) and verified for specificity. And siRNA sequences for core genes (*TK1*, *ATP5F1B*, *IMPDH*) and si-NC, synthesized with 2'-O-methyl modification and no predicted off-target sites.

|  | **Target gene/siRNA type** | **Gene name** | **Forward primer (5′→3′)** | **Reverse primer (5′→3′)** | **Pathway** |
| --- | --- | --- | --- | --- | --- |
| 1 | TK1 | TK1 | GGCATCCTGAAGATGCTGAA | AGGTGGTGGTGATGGTGTAG | Yes |
|  | TK1  (core gene) | si-TK1 | GCAUCCUGAAGAUGCUGAAdTdT | UUCAGCAUCUUCAGGAUGCdTdT |  |
| 2 | ATP5F1B | ATP5F1B | GCTGGTGCTGAAGAAGATCG | TCGATGGTGGTGGTGATGTC | Yes |
|  | ATP5F1B  (core gene) | si-ATP5F1B | GCUGGUGCUGAAGAAGAUCGdTdT | CGAUCUUCUUCAGCACCAGCdTdT |  |
| 3 | IMPDH | IMPDH | GCTGTGGATGAAGGTGAAGC | TCGGTGATGGTGGTGAAGTC | Yes |
|  | IMPDH  (core gene) | si-IMPDH | GCUGUGGAUGAAGGUGAAGCdTdT | GCUUCACCUUCAUCCACAGCdTdT |  |
| 4 | Agmatine sulfate | Agmatine sulfate | CAGCAGTTGGAGAACGTGAA | GTCATAGTCCTCGGTCACGT | NA |
|  | Agmatine sulfate (core gene) | si-Agmatine sulfate | GCUCCAACUUCAGAAGCUAdTdT | UAGCUUCUGAAGUUGGAGCdTdT |  |
| 5 | Biotin | Biotin | TGCAGTGGAAGATGGCTTCA | CTGGGTGTTGTCGATGGTGT | NA |
|  | Biotin  (core gen) | si-Biotin | GCAUGAGCUUCAAGUGCAUdTdT | AUGCACUUGAAGCUCAUGCdTdT |  |
| 6 | Choline | Choline | TGTGCTGGATGAAGTGGAGA | AGGTGATGGTGAGGGTGATG | NA |
|  | Choline  (core gen) | si-Choline | GGCUUCAGUACAAGGAGAUdTdT | AUCUCCUUGUACUGAAGCCdTdT |  |
| 7 | Guanine | Guanine | GAGATGGCAGTGGATGAGTT | CTGGTAGTCTTCAGGCACTC | NA |
|  | Guanine  (core gen) | si-Guanine | GCUCAUGAACGAGUUCAAUdTdT | AUUGAACUCGUUCAUGAGCdTdT |  |
| 8 | Oxypurinol | Oxypurinol | TGGATGAGGCTGAGAAGGTG | GGTGATGGTGGTGTCTGTGT | NA |
|  | Oxypurinol  (core gen) | si-Oxypurinol | GAGCUGAAGGAGAUCCAUUdTdT | AAUGGAUCUCCUUCAGCUCdTdT |  |
| 9 | Glycocholic acid | Glycocholic acid | CTTCGGCTACATCGAGTGC | GGTGATGATGTAGCGGTTGT | NA |
|  | Glycocholic acid (core gen) | si-Glycocholic acid | GCUACAUCGAGUGCAUCAAdTdT | UUGAUGCACUCGAUGUAGCdTdT |  |
| 10 | Capryloylglycine | Capryloylglycine | TGGAGCAGTTCGAGAAGGTG | CTTGATGGTGGTGAGGTCGT | NA |
|  | Capryloylglycine  (core gen) | si-Capryloylglycine | GCAGUUCGAGAAGGUGCUUAUdTdT | AUAAGCACCUUCUCGAACUGCdTdT |  |
| 11 | N-Acetyl-L-tyrosine | N-Acetyl-L-tyrosine | CTGAGCTGAAGATCCGTGTG | AGGTGATGGTGAAGGTCAGG | NA |
|  | N-Acetyl-L-tyrosine (core gen) | si-N-Acetyl-L-tyrosine | CUGAGCUGAAGAUCCGUGUGdTdT | CACACGGAUCUUCAGCUCAGdTdT |  |
| 12 | Oleoyl ethanolamide | Oleoyl ethanolamide | GATGGCTTCTACCGTGAGCA | TCGATGGTGATGGTGAGGTT | NA |
|  | Oleoyl ethanolamide  (core gen) | si-Oleoyl ethanolamide | GCUUCUACCGUGAGCAGUUdTdT | AACUGCUCACGGUAGAAGCdTdT | NA |
| 13 | Negative control | si-NC | UUCUCCGAACGUGUCACGUTT | ACGUGACACGUUCGGAGAATT |  |
|  | *β*-actin | *β*-actin | GCTGTCTTCCCTGGAATCGT | CAGGACTCCATGCCCAGGA |  |
