# Supplementary material for: Transcriptomic and Metabolomic Profiling Identifies a Core Gene–Metabolite Axis Driving African Swine Fever Virus Replication in the Soft Tick Ornithodoros lahorensis
Source: FASEB J. 2026 Aug 31;40(17):e72256. doi: 10.1096/fj.202602001RR (PMC13528556; doi:10.1096/fj.202602001RR)
Supplement: Supplementary file 7 [file FSB2-40-e72256-s007.docx]

****Supplementary Figure S2. Volcano plots of differentially expressed metabolites (left) and genes (right) across three pairwise comparisons.****

**Note: Upregulated molecules = red dots, downregulated = blue dots, non-significant = gray. Triangle = VIP<1, circle = VIP≥1 for metabolome data.**

| **Comparegroup** | **Num_Diff_metabolite** | **Num_Diff_gene** |
| --- | --- | --- |
| Q5-VS-Q1 | 891 | 42586 |
| Q4-VS-Q1 | 860 | 16117 |
| Q5-VS-Q4 | 834 | 42892 |
