# Supplementary material for: Transcriptomic and Metabolomic Profiling Identifies a Core Gene–Metabolite Axis Driving African Swine Fever Virus Replication in the Soft Tick Ornithodoros lahorensis
Source: FASEB J. 2026 Aug 31;40(17):e72256. doi: 10.1096/fj.202602001RR (PMC13528556; doi:10.1096/fj.202602001RR)
Supplement: Supplementary file 8 [file FSB2-40-e72256-s008.docx]

**Supplementary Table S4. Broad-spectrum qRT-PCR validation of 40 randomly selected non-core differentially expressed transcripts.**

Note: RNA-seq log₂(fold change) values and qRT-PCR relative expression fold changes (Q5 vs Q1) are listed for each randomly sampled DEG. Concordance between RNA-seq and qRT-PCR up/downregulation trends was calculated to quantify the global reliability of transcriptomic differential expression outputs.

| **No.** | **Transcript/Gene ID** | **RNA-seq log₂(Fold Change) (Q5 vs Q1)** | **qRT-PCR Relative Expression Fold Change (Q5 vs Q1)** | **Concordance** |
| --- | --- | --- | --- | --- |
| 1 | DEG_UP_1 | 3.31 | 8.03 | Yes |
| 2 | DEG_UP_2 | 2.11 | 3.84 | Yes |
| 3 | DEG_UP_3 | 3.63 | 13.26 | Yes |
| 4 | DEG_UP_4 | 4.14 | 14.72 | Yes |
| 5 | DEG_UP_5 | 2.59 | 4.89 | Yes |
| 6 | DEG_UP_6 | 1.92 | 3.79 | Yes |
| 7 | DEG_UP_7 | 1.29 | 2.15 | Yes |
| 8 | DEG_UP_8 | 3.34 | 10.31 | Yes |
| 9 | DEG_UP_9 | 1.93 | 3.95 | Yes |
| 10 | DEG_UP_10 | 3.87 | 11.74 | Yes |
| 11 | DEG_UP_11 | 3.86 | 15.67 | Yes |
| 12 | DEG_UP_12 | 2.32 | 4.31 | Yes |
| 13 | DEG_UP_13 | 4.36 | 19.19 | Yes |
| 14 | DEG_UP_14 | 1.51 | 2.39 | Yes |
| 15 | DEG_UP_15 | 4 | 16.66 | Yes |
| 16 | DEG_UP_16 | 3.86 | 15.85 | Yes |
| 17 | DEG_UP_17 | 2.97 | 9.32 | Yes |
| 18 | DEG_UP_18 | 2.45 | 5.58 | Yes |
| 19 | DEG_UP_19 | 3.94 | 16.08 | Yes |
| 20 | DEG_UP_20 | 4.04 | 4.93 | No |
| 21 | DEG_DOWN_21 | -2.17 | 0.18 | Yes |
| 22 | DEG_DOWN_22 | -3.75 | 0.07 | Yes |
| 23 | DEG_DOWN_23 | -4.24 | 0.05 | Yes |
| 24 | DEG_DOWN_24 | -4.17 | 0.05 | Yes |
| 25 | DEG_DOWN_25 | -2.4 | 0.18 | Yes |
| 26 | DEG_DOWN_26 | -3.28 | 0.09 | Yes |
| 27 | DEG_DOWN_27 | -3.62 | 0.1 | Yes |
| 28 | DEG_DOWN_28 | -2.36 | 0.2 | Yes |
| 29 | DEG_DOWN_29 | -3.94 | 0.07 | Yes |
| 30 | DEG_DOWN_30 | -3.96 | 0.06 | Yes |
| 31 | DEG_DOWN_31 | -1.23 | 0.45 | Yes |
| 32 | DEG_DOWN_32 | -2.66 | 0.17 | Yes |
| 33 | DEG_DOWN_33 | -1.72 | 0.34 | Yes |
| 34 | DEG_DOWN_34 | -3.74 | 0.06 | Yes |
| 35 | DEG_DOWN_35 | -3.46 | 0.08 | Yes |
| 36 | DEG_DOWN_36 | -3.8 | 0.08 | Yes |
| 37 | DEG_DOWN_37 | -1.61 | 0.3 | Yes |
| 38 | DEG_DOWN_38 | -2.34 | 0.19 | Yes |
| 39 | DEG_DOWN_39 | -1.48 | 0.35 | Yes |
| 40 | DEG_DOWN_40 | -3.63 | 0.24 | No |
| Total Concordance Rate | | | 38/40 (95.0%) | |
